# Nasotracheal Intubation in Extreme Prematurity and Traumatic Brain Injury: A Cautionary Tale

**DOI:** 10.1155/crpe/3005450

**Published:** 2025-10-03

**Authors:** Cíntia Junges, Jarred Garfinkle, Maryam Oskoui

**Affiliations:** ^1^Centre for Outcomes Research and Evaluation, Research Institute of the McGill University Health Center, Montreal, Quebec, Canada; ^2^Department of Pediatrics, McGill University, Montreal, Quebec, Canada; ^3^Department of Neurology & Neurosurgery, McGill University, Montreal, Quebec, Canada

**Keywords:** cerebral damage, cribriform plate, long-term outcomes, nasotracheal intubation, practice review

## Abstract

We report a case of a male neonate delivered urgently via cesarean at 27 weeks of gestation for placenta abruption who was apneic at birth and needed endotracheal intubation. Traumatic nasal intubation with injury to the cribriform plate and tract through the right cerebral hemisphere occurred. Now at 10 years of age, he has left hemiplegic cerebral palsy, autism spectrum disorder, intellectual disability, and externalizing disruptive behavior. This case highlights a potential complication of nasal intubation in preterm infants and the importance of considering this in choosing the route of intubation in preterm infants.

## 1. Introduction

There is no evidence-based consensus on the preferred route of endotracheal intubation in preterm infants. Supporters of the oral route argue that oral intubation is easier to perform and less traumatic to the infant. Those who prefer the nasal route state that nasal tubes have more stability with a lower rate of accidental extubation than oral tubes [1, 2].

Traumatic complications of nasotracheal intubation with perforation of the lamina cribrosa in preterm infants have been reported [2–5]. Here, we present a complication of nasotracheal intubation of an extremely preterm infant that occurred at birth and his long-term follow-up to 10 years of age.

## 2. Case Report/Case Presentation

### 2.1. Perinatal History

Our patient was born to a healthy 33-year-old mother with normal fetal ultrasounds. She evolved with a short cervix, premature rupture of membranes, and vaginal bleeding. Emergent cesarean section for placenta abruption was performed at 27 weeks of gestation. She had received antibiotic therapy for premature rupture of membranes and completed a betamethasone course before the delivery.

### 2.2. Neonatal Course

**Figure 1:**
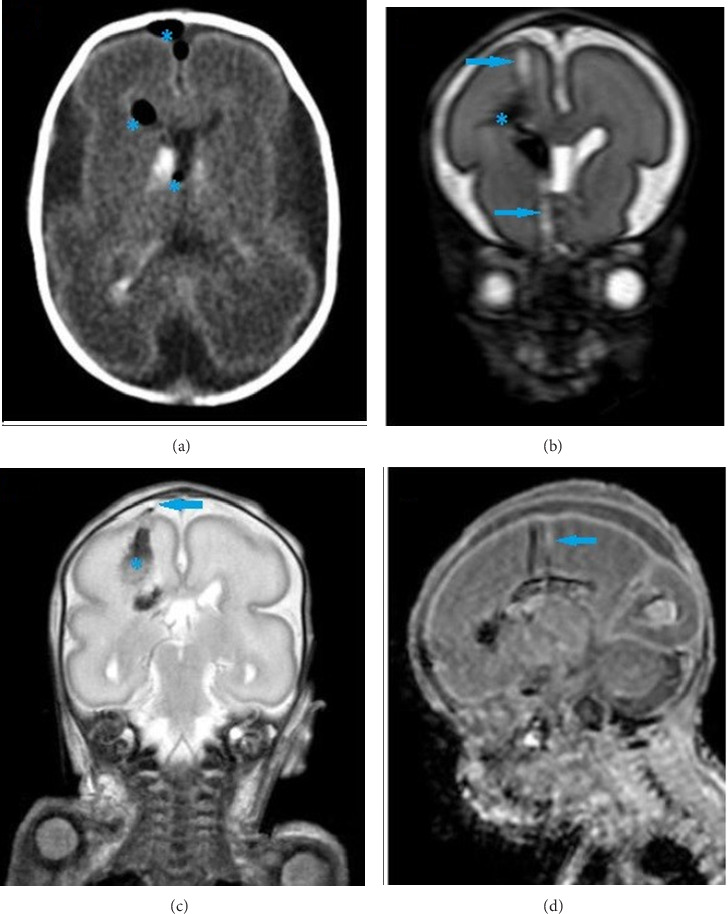
CT (a) highlights pneumocephalus (asterisks) related to the occult fracture at the skull base. Brain MRI images T2-weighted (b and c) and T1-weighted (d) demonstrate traumatic tract (arrows) into the right cerebral hemisphere extending from the ethmoid air cells to the upper parietal cortex with posttraumatic right grade 4 intraventricular hemorrhage (asterisk).

The male preterm infant was apneic at birth. Intubation through the right nostril was first attempted. The endotracheal tube (ETT) went in 5 cm but was not visible in the oropharynx, and bloody serous fluid was seen upon removing the ETT. Upon the second attempt, the ETT could not pass the left nostril. Finally, there was successful oral intubation, and he began to breathe spontaneously at 10 min of life. The Apgar scores were 1, 4, and 7 at 1, 5, and 10 min. Birth weight (980 g), length (37.5 cm), and head circumference (24.5 cm) were appropriate for gestational age. Computerized tomography and magnetic resonance imaging (MRI) of the brain in the first days of life diagnosed an injury to the cribriform plate and a tract through the right cerebral hemisphere with intracranial and intraventricular hemorrhage secondary to traumatic nasal intubation (Figure 1). He subsequently received antibiotic therapy for 21 days. A septal deviation was also diagnosed.

### 2.3. Long-Term Follow-Up

**Figure 2:**
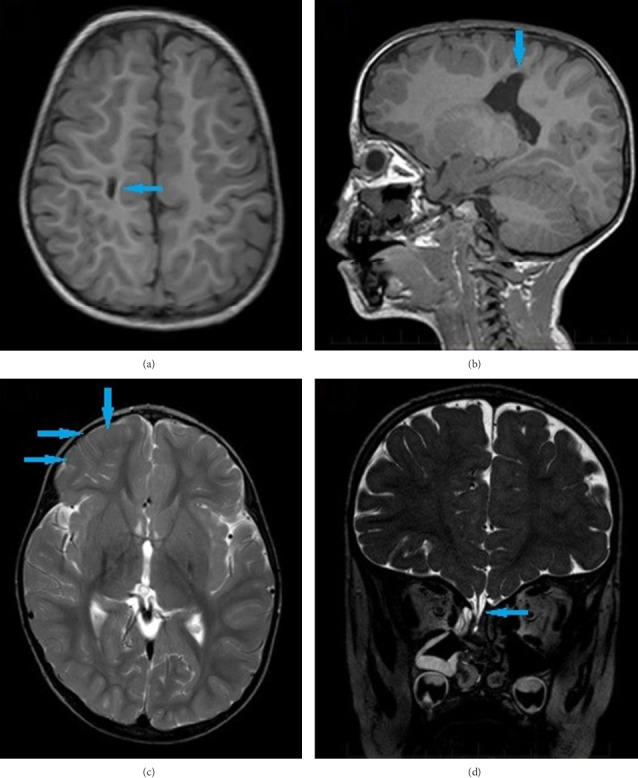
T1-weighted images (a and b) evidence motor pathway injury (arrows). T2-weighted image in (c) shows sequelae of prefrontal cortical and subcortical injury (arrows). 3D DRIVE T2-weighted image in (d) highlights the herniation of the rectus gyrus of the right frontal lobe through the base skull defect (arrow).

He was discharged at approximately 3 months of life (40 weeks corrected age). At that moment, no neurological concerns were reported. At 9 months, he underwent skull base repair due to persistent cerebrospinal fluid leakage. Brain MRI at 3 years showed right frontoparietal lobe abnormalities with stable frontal lobe herniation (Figure 2). Now at 10 years of age, he has left hemiplegic cerebral palsy with Gross Motor Function Classification System Level II [6], autism spectrum disorder, moderate intellectual disability, and externalizing disruptive behaviors (aggressivity, impulsivity, and hyperactivity).

## 3. Discussion/Conclusion

We presented a case of lamina cribrosa perforation in a preterm infant from nasotracheal intubation with consequent formation of a tract to the right cerebral hemisphere with motor pathway and prefrontal cortical and subcortical injuries. Long-term follow-up demonstrated significant morbidity with clinical–anatomical correlation, with motor, neuropsychiatric, and cognitive impairments.

In the newborn, the anterior skull base is predominantly cartilaginous and thus fragile. The ossification of the cribriform plate is poorly described in the literature but probably begins during the first postnatal month and progresses during the first year of life [5, 7]. No evidence supports the superiority of orotracheal intubation over nasotracheal intubation [1]. To our knowledge, seven previous case reports describe cribriform plate perforation by nasotracheal intubation in preterm infants [2–5, 8], highlighting the importance being aware of the fragility of the posterior nasal structures before performing this delicate procedure.

Different from the other case reports, our report provides the longest follow-up of the patient. It is known that cognitive, behavioral, and motor deficits are potential sequelae in preterm children, even in the absence of overt brain injury [4]. However, our case illustrates clinical–anatomical correlation supporting the relationship between the traumatic right motor pathway and frontal injuries that occurred at birth and the developmental sequelae observed 10 years later. The left hemiplegic cerebral palsy can be explained by the existence of an injury to the right motor pathway, while the psychiatric and cognitive sequelae may be associated with the right prefrontal injury [9].

The purpose of this case report is to emphasize the possibility of significant long-term morbidity resulting from a complication of nasotracheal intubation in preterm infants. The theoretical benefits of nasotracheal intubation must be weighed against the potential for traumatic brain injury and neurodevelopmental sequelae. In our neonatal intensive care unit, nasotracheal intubation is no longer practiced. To ensure safe insertion of nasal ETTs, the tube should be removed if resistance is felt. In addition, others have suggested to avoid rotating the tube during insertion and to use a small feeding tube to act as a guide over which the ETT can be gently slid [2].

## Data Availability

The data that support the findings of this study are available from the corresponding author upon reasonable request.
